# Facial Neuralgia

**Published:** 1844-11-16

**Authors:** 


### FACIAL NEURALGIA.

In the Lancette Francaise, is detailed the case of a tobacconist who presented himself a short time back at the consultation of M. Velpeau, suffering intensely for eleven years with facial neuralgia. He bore unequivocal marks of blisters, moxas, and setons having been applied in great numbers, and had moreover taken a prodigious quantity of Meglin's pills. Some surgeon had attempted a cure of the complaint by dividing one of the branches of the tri-facial nerve and he had extracted (as has often happened) all the teeth of the upper jaw on the affected side. After examining him it was found impossible to devise any method that had not already been fruitlessly employed, when M. Pajol, a young physician, thought of asking him if he had ever been the subject of lues venerea. He admitted that he had a bubo, for which he had not undergone a specific treatment. Upon this intimation, M. Velpeau prescribed for him a treatment with the proto-ioduret of mercury. By the employment of this remedy the man recovered, and did not again suffer from neuralgia. It is very probable that many osteoscopic pains are nothing else than syphilitic neuralgia, particularly when it is remembered that they sometimes yield in a miraculous manner to the application of several blisters to the part affected, as M. Ricord has fully established; and that, where the blister has not succeeded, a deep incision over the seat of pain has answered the same intention. The efficacy of repeated blisters against neuralgia is well-known, and Dr. Marchal, of Calvi, has shown that in certain cases of cranial neuralgia, a deep incision of the parts has had a most prompt and sedative effect on the pains.—*Lond. Med. Times.*

